# Green AI – A multidisciplinary approach to sustainability

**DOI:** 10.1016/j.ese.2025.100536

**Published:** 2025-01-26

**Authors:** Jerry Huang, Suchi Gopal

**Affiliations:** a John A. Paulson School of Engineering and Applied Sciences, Harvard University, USA; b Department of Earth and Environment, Boston University, USA

**Fig. 1:**
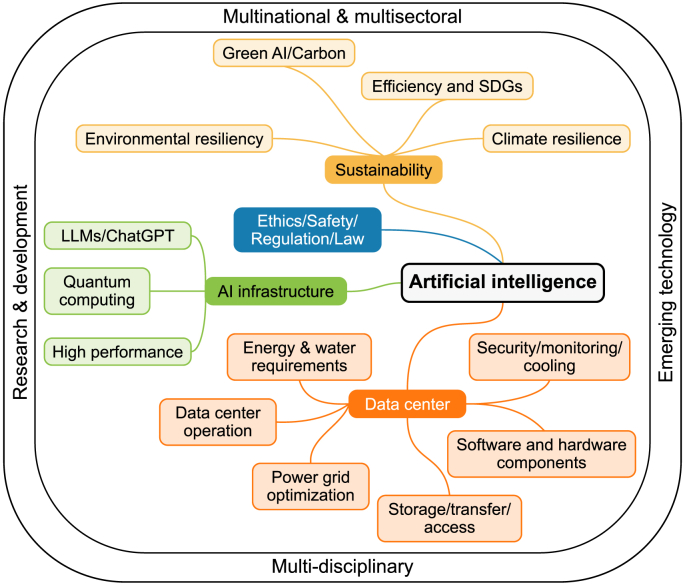
Framework for sustainable AI: multidisciplinary and multisectoral integration.

The rapid growth of artificial intelligence (AI) technologies has sparked concerns about their environmental impact, particularly regarding electricity consumption and the challenges this poses to climate change mitigation efforts. As AI models and data centers continue to expand, their escalating energy demands risk outpacing power infrastructure development, potentially leading to a “power grid crisis.” Data centers, which are at the core of AI operations, consume vast amounts of electricity, and their energy-intensive nature could undermine progress toward climate goals (see Fig. 1).

A recent white paper by the Green AI Institute highlights the need for a sustainability framework that incentivizes data centers to meet environmental criteria. This urgency is reinforced by Bloomberg Intelligence projections, which estimate that by 2030, data centers and AI-related infrastructure could account for up to 17 % of United States electricity consumption [1]. A significant portion of this demand stems from the energy-intensive processes required to train and deploy large-scale AI models, placing further strain on existing energy systems.

Responding to these exacerbating pressures, efforts to align AI advancements with sustainability goals are gaining traction globally. One such initiative that drives this conversation is the White Paper on Global Artificial Intelligence Environmental Impact by the Green AI Institute, launched at the Green AI Summit, a global dialogue hosted by the Green AI Institute on October 26, 2024 [2]. The White Paper provided a comparative analysis of AI-related environmental policies across the United States, European Union, and China, and introduced the Green AI Index, a standardized set of metrics to evaluate the environmental impact of AI technologies, including factors such as carbon emissions, energy, and water consumption. This tool is designed to foster transparency and accountability, encouraging sustainable global AI practices. The Green AI Institute also recently published the Journal of AI and Sustainability and a white paper on the Carbon Neutrality of Chinese Manufacturing. These publications provided a platform for disseminating research, emphasizing AI's role in transforming energy-intensive industries through enhanced efficiency and reduced emissions. A call to action encouraged collaboration between industries and academia to share knowledge and best practices globally.

One of the key barriers to creating more sustainable AI is power grid-AI integration—to effectively coordinate between data centers and power grids, and experts are exploring how AI can optimize grid operations to reduce peak energy demand. Insights were provided on AI's ability to dynamically manage facility energy loads, highlighting its potential to drive sustainability. The growing global demand for computational power was juxtaposed with the urgent need for innovative energy sources to support this growth responsibly.

Recent efforts drive cutting-edge computing technologies designed to mitigate AI infrastructure's environmental footprint, such as quantum and high-performance computing, which have a high potential to reduce energy intensity in computation-heavy industries. Experts also discussed eco-friendly design principles for AI hardware aimed at minimizing waste and maximizing efficiency. These sustainable computing practices must be successfully integrated into national and international energy policies to drive collective action.

One innovative technology that demonstrates AI's role in climate change mitigation is the SeaWARRDD system [3]. This project showcased advanced AI-enabled sensors that track atmospheric and oceanic changes, enhancing hurricane forecasting. The session further highlighted AI's integration in creating predictive models for disaster management, illustrating its transformative potential to reduce environmental risks.

A factor that significantly impacts AI's role in achieving the United Nation's Sustainable Development Goals (SDGs) is the strategic placement of data centers, which are central to AI's energy consumption. It is vital for data centers to be situated near renewable energy sources, such as wind and solar power, and ensure access to water resources for cooling. Given data centers' high water consumption, AI-driven solutions for precision farming and resource optimization were identified as critical tools to reduce environmental degradation. The discussion also highlighted AI's ability to manage urban energy and water systems, reducing cities' ecological footprints. Innovations in AI-powered waste management and material recycling were proposed as pathways toward a zero-waste model.

Beyond practical applications, it is also necessary to acknowledge that ethical considerations and regulatory frameworks are central to the third panel on AI Governance for Sustainability. Discussions focused on ensuring AI systems remain accessible and equitable across regions, emphasizing the need for unified international standards to regulate AI's environmental impacts. Participants explored AI's dual-edged nature, advocating for safeguards to prevent ecological harm while maximizing its potential for good.

Simultaneously, from AI-driven renewable energy platforms to the discovery of biodegradable materials, startups developed by young entrepreneurs were recognized as pivotal drivers of sustainable AI solutions. Fostering creativity and leadership among young innovators was a recurring theme, highlighting their critical role in advancing sustainability.

In addition to these advancements, strong leadership is needed with holistic policies and global collaboration to align AI with sustainable development goals. Policymakers were urged to integrate technological and societal goals, creating a unified framework for sustainable AI adoption. Leaders in the field identify cross-sector partnerships as vital to scaling AI innovations without exacerbating environmental degradation.

Ultimately, to achieve green, responsible AI, it is necessary to scale renewable energy integration within AI systems, establish global regulatory frameworks to address AI's ecological footprint and expand education and public awareness about AI's role in advancing sustainability.
